# Intermuscular coherence between homologous muscles during dynamic and static movement periods of bipedal squatting

**DOI:** 10.1152/jn.00231.2020

**Published:** 2020-08-20

**Authors:** Rouven Kenville, Tom Maudrich, Carmen Vidaurre, Dennis Maudrich, Arno Villringer, Patrick Ragert, Vadim V. Nikulin

**Affiliations:** ^1^Institute for General Kinesiology and Exercise Science, Faculty of Sports Science, University of Leipzig, Leipzig, Germany; ^2^Max Planck Institute for Human Cognitive and Brain Sciences, Department of Neurology, Leipzig, Germany; ^3^Centre for Cognition and Decision Making, Institute for Cognitive Neuroscience, National Research University Higher School of Economics, Moscow, Russian Federation; ^4^Neurophysics Group, Department of Neurology, Charité-University Medicine Berlin, Campus Benjamin Franklin, Berlin, Germany; ^5^Department of Statistics, Informatics and Mathematics, Public University of Navarre, Pamplona, Spain; ^6^Machine Learning Group, Faculty of EE and Computer Science, TU Berlin, Berlin, Germany; ^7^MindBrainBody Institute at Berlin School of Mind and Brain, Charité-Universitätsmedizin Berlin and Humboldt-Universität zu Berlin, Germany; ^8^Clinic for Cognitive Neurology, University Hospital Leipzig, Leipzig, Germany

**Keywords:** bipedal squat, compound movement, intermuscular coherence, neural oscillations

## Abstract

Coordination of functionally coupled muscles is a key aspect of movement execution. Demands on coordinative control increase with the number of involved muscles and joints, as well as with differing movement periods within a given motor sequence. While previous research has provided evidence concerning inter- and intramuscular synchrony in isolated movements, compound movements remain largely unexplored. With this study, we aimed to uncover neural mechanisms of bilateral coordination through intermuscular coherence (IMC) analyses between principal homologous muscles during bipedal squatting (BpS) at multiple frequency bands (alpha, beta, and gamma). For this purpose, participants performed bipedal squats without additional load, which were divided into three distinct movement periods (eccentric, isometric, and concentric). Surface electromyography (EMG) was recorded from four homologous muscle pairs representing prime movers during bipedal squatting. We provide novel evidence that IMC magnitudes differ between movement periods in beta and gamma bands, as well as between homologous muscle pairs across all frequency bands. IMC was greater in the muscle pairs involved in postural and bipedal stability compared with those involved in muscular force during BpS. Furthermore, beta and gamma IMC magnitudes were highest during eccentric movement periods, whereas we did not find movement-related modulations for alpha IMC magnitudes. This finding thus indicates increased integration of afferent information during eccentric movement periods. Collectively, our results shed light on intermuscular synchronization during bipedal squatting, as we provide evidence that central nervous processing of bilateral intermuscular functioning is achieved through task-dependent modulations of common neural input to homologous muscles.

**NEW & NOTEWORTHY** It is largely unexplored how the central nervous system achieves coordination of homologous muscles of the upper and lower body within a compound whole body movement, and to what extent this neural drive is modulated between different movement periods and muscles. Using intermuscular coherence analysis, we show that homologous muscle functions are mediated through common oscillatory input that extends over alpha, beta, and gamma frequencies with different synchronization patterns at different movement periods.

## INTRODUCTION

Everyday life activities comprise not only isolated movements but also compound whole body movements such as walking, climbing stairs, and standing (Casale et al. 2011). Isolated movements are usually studied under laboratory settings, with the degrees of freedom of such movements being limited. In contrast, whole body movements require extensive control of many muscle groups of the upper and lower extremities. Understanding how the central nervous system asserts control over such movements has important practical implications, as disorders of the motor system are particularly detrimental and costly to patients (Mozaffarian et al. 2016; Singh et al. 2014).

A commonly employed method to examine motor control characteristics is intermuscular coherence (IMC), i.e., the analysis of linear dependencies between two electromyography (EMG) recordings at a certain frequency (Gross et al. 2002). Using IMC, it is possible to investigate common synaptic input to motor neuron pools across muscles in humans noninvasively (Dideriksen et al. 2018). IMC is associated with cortical and spinal mechanisms (Boonstra 2013; Boonstra and Breakspear 2012; Grosse and Brown 2003) and was previously used to demonstrate functional binding between muscles (Laine and Valero-Cuevas 2017). Motor-relevant oscillatory components are at alpha (∼8–12 Hz), beta (∼13–30 Hz), and gamma (∼>30 Hz) frequencies. Synchronized oscillations at alpha frequencies are present during the activity of different muscles during uni- and bimanual motor control tasks of upper (de Vries et al. 2016; McAuley et al. 1997) and lower extremities (Boonstra et al. 2008, 2015). Beta band oscillations have been observed in functionally related muscles (Boonstra 2013; Boonstra and Breakspear 2012; Kilner et al. 1999) and were originally thought to reflect efferent origin (Brown et al. 1999), although recent research indicates a more complex efferent-afferent feedback loop as the potential source for the presence of these oscillations (Witham et al. 2010, 2011). Gamma band IMC has also been observed during numerous movements and is most prominently related to more compound, dynamic movements (De Marchis et al. 2015; Mohr et al. 2015, 2018). There is considerable overlap between the neuronal mechanisms of beta and gamma band IMC. Still, although both beta and gamma IMC reflect corticomuscular drive (Boonstra 2013; Farmer 1998; Mima and Hallett 1999), beta IMC is commonly observed during isolated muscle control (Boonstra 2013; Boonstra and Breakspear 2012; Farmer 1998; McManus et al. 2016; Mima and Hallett 1999), and gamma IMC is associated with integrative processes in the coordination of compound and/or novel movement sequences (Chang et al. 2012; De Marchis et al. 2015; Mohr et al. 2018; Omlor et al. 2007).

To facilitate the transition between theory and applications in motor control research, the studied movements need to be naturalistic, i.e., closely related to everyday life activities. On a whole body level, everyday life activities can be divided into unilaterally alternating movements such as walking and climbing stairs on the one hand and bilateral movements such as picking up loads, sitting, and standing up on the other. Both alternating and bilateral movement sequences show large physiological differences, e.g., distinct cortical activation profiles (Kapreli et al. 2006; Noble et al. 2014) and different inhibition patterns (Aune et al. 2013). Similarly, the degree of fatigue of individual muscles (Jakobi and Chilibeck 2001), the contribution of postural muscles (Janzen et al. 2006; Magnus and Farthing 2008), especially in movements of the lower limbs (Magnus and Farthing 2008), and the targeting of muscle fiber types (Buckthorpe et al. 2013; Koh et al. 1993) differ between bilateral and alternating movements. Furthermore, recent research suggested potentially increased neuroplasticity in bilateral compared with alternating movements (Whitall et al. 2011), which may aid in the facilitation of neurorehabilitative strategies (Cauraugh et al. 2010; Cauraugh and Summers 2005).

With this in mind, the bipedal squat (BpS) is a valuable extension to compound motor control research, as BpS comprises bilateral movement patterns of everyday life (Nelson et al. 2002). A recent study by Mohr et al. (2015) examined unilateral intermuscular interactions during BpS using IMC. The authors observed IMC between a nonhomologous muscle pair of the thighs during BpS performance and found IMC to be present at frequencies ranging from 15 to 80 Hz (Mohr et al. 2015). However, two important aspects of BpS motor control remain unexamined and should be assessed to gain a better understanding of BpS motor control. First, Mohr and colleagues (Mohr et al. 2015) did not analyze IMC between homologous muscles. During BpS, pairs of homologous upper and lower body muscles jointly achieve bipedal and postural stability and enable bilateral execution of BpS (Thiele et al. 2015). Such homologous coordination is essential to enable the successful execution of fundamental movements of everyday life (Kang et al. 2019; Seidler et al. 2010). Although previous studies have investigated IMC between homologous muscles (Boonstra et al. 2008, 2009), common oscillatory input of principal homologous muscle pairs of the upper body and homologous muscle pairs of the lower body has not been studied during BpS. We therefore aim to extend previous findings and to examine common synaptic input between principal homologous muscle pairs to uncover bilateral aspects of BpS motor control. Second, the extent to which IMC is modulated between static (isometric) and dynamic (eccentric and concentric) movement periods during BpS is unclear. Movement periods, i.e., eccentric (ECC), isometric (ISO), and concentric (CON) periods, pose different challenges on acting muscles, resulting in muscles functioning in distinct roles between periods. It is therefore crucial to analyze individual modulations of central nervous involvement during each period of BpS. Although IMC is most frequently analyzed during isometric movement periods (Baker et al. 1999; Kilner et al. 1999; Semmler et al. 2013), there have been studies investigating IMC during dynamic movements. For instance, IMC between different recording sites of one muscle was most pronounced during ECC compared with ISO and CON during contractions of first dorsal interosseous muscles (FDI) (Semmler et al. 2006) and gastrocnemius (von Tscharner 2014). In general, functional relations of frequency band-specific IMC and movement periods have been examined in previous studies. Beta IMC has been prominently observed during static movement periods (ISO) (Kilner et al. 1999; Reyes et al. 2017), whereas gamma IMC was shown to be increased during dynamic contractions (CON and ECC) when compared with isometric contractions (Semmler et al. 2002; von Tscharner 2014). Although movement period-related modulations of alpha IMC have rarely been studied, evidence suggests stable behavior of alpha IMC between movement periods (Nguyen et al. 2017).

Based on the aforementioned findings, we hypothesize that bilateral control of principal homologous muscle pairs during BpS is in part achieved through common input into those muscle pairs. Accordingly, we hypothesize to find IMC in motor-relevant frequency bands alpha, beta, and gamma across principal homologous muscle pairs in BpS. Furthermore, we hypothesize that there is a clear distinction between IMC magnitudes during isometric (ISO) and dynamic (ECC and CON) movement periods based on different muscle functions between movement periods. More specifically, based on previous evidence, we expect to identify highest beta IMC during ISO and highest gamma IMC during ECC, while we do not expect movement period-related changes in alpha IMC magnitude.

## MATERIALS AND METHODS

### 

#### Participants.

We recruited 11 healthy, male participants [age: 27.9 ± 5.1 yr (mean ± SD)] in the present study. The study was endorsed by the local committee of the Medical Faculty at the University of Leipzig (ref. no. 466/17-ek). We recruited only male participants to avoid variance due to possible gender-related differences in brain structure and function (Grabowska 2017) as well as differences in activation profiles during squats (Graci et al. 2012; Hale et al. 2014; Mehls et al. In press). Before participation, all participants provided their written, informed consent to take part in the experiments following the Helsinki Declaration. To minimize the risk of injury, participants were excluded in case any of the following exclusion criteria were present: neurological/psychiatric disease; intake of centrally acting drugs; caffeine or alcohol intake 24 h before the experiment; acute, chronic, and/or inadequately regenerated pathologies of the knee joint, the ankle joints, and/or the spine. Also, we chose to exclude participants with regular sports activity (>3 h/wk). The rationale for this was that previous studies had demonstrated that sports competence influences coherence, which would impact analyses and interpretation of results (Ushiyama et al. 2010).

#### Behavioral task (bipedal squat).

The following descriptions of our experimental setup, as well as all acquired behavioral data, are based on a previous study we conducted (Kenville et al. 2020). For details, please refer to the respective article. Still, we mention below the most important aspects. Initially, participants were instructed concerning the correct execution of BpS. All participants were advised to plant their feet and execute BpS without raising their heels during force exertion. Additionally, each participant was instructed to keep a slight lumbar lordosis during BpS, as well as to keep their head aligned with the spine. During BpS, arms remained in an extended, relaxed position beside the body. A short (3 min) warm-up program of controlled repetitions of dynamic squats without additional load preceded the actual measurements. Here, participants were instructed to execute BpS in a manner that focused on the aforementioned key aspects of correct movement execution, i.e., *1*) planting of the feet and *2*) slight lumbar lordosis. For a repetition, the participants started with their legs fully extended at the beginning of the eccentric movement periods (ECC), squatted until a knee angle of 95° was reached (the squatting depth was determined employing a protractor), held this position during the isometric period (ISO), and then extended their legs once again during the concentric movement period (CON).

#### Procedure.

In total, 40 trials of BpS were completed. The experiments were conducted in blocks of 10 repetitions, with break periods of 3:30 min separating each block to avoid possible cumulative consequences due to peripheral fatigue. As mentioned, each squatting repetition was split into three 5-s movement periods (ECC, muscles are being stretched as they contract; ISO, muscles keep their length while contracting; CON, muscles shorten throughout the contraction), resulting in three conditions altogether. A break period of 30 s succeeded each repetition (ECC-ISO-CON). All periods were visually initiated on a standard PC monitor running Presentation 16.5 software (NeuroBehavioral Systems, Albany, NY). The participants were all naive to the task of BpS. For an overview of average EMG activity for all muscles and periods, please see Supplemental Figure S1 (all Supplemental figures are available at https://doi.org/10.6084/m9.figshare.12618929).

#### EMG recordings.

We used a wireless Desktop Transmission System (NORAXON Inc., Scottsdale, AZ) to measure surface EMG signals from four homologous muscles mainly active during squat execution. Bipolar surface electrodes (Ag/AgCl; diameter: 1 cm) were mounted bilaterally on four homologous muscles [vastus lateralis (VL), vastus medialis (VM), tibialis anterior (TA), erector spinae (ES)] in accordance to SENIAM electrode position recommendations (Hermens et al. 2000). A fixed interelectrode distance (2 cm) was maintained throughout the recordings. Each participant's skin was shaved to remove hair around the electrode area and was exfoliated. Double-sided adhesive tape was used to attach all transmitters mounted in the proximity of the electrodes. The EMG sensors were positioned in a parallel alignment relative to the muscle fibers. Furthermore, the display of each movement period onset was synchronously triggered on a PC screen to enable synchronizing movement onsets. In particular, the participants were presented with a standardized white cross on a screen before movement onset. Three seconds before the start of the initial movement period (ECC) the cross turned green, indicating that the participant should prepare for movement onset. The following movement periods were precisely initiated by a time-exact presentation of their abbreviations on the screen. We recorded data of 8 channels with a sampling frequency of 3,000 Hz, an input impedance of the amplifier >100 MΩ, bandpass filtering in the frequency range of 10–500 Hz, common-mode rejection (CMRR) >100 dB, a gain of 500.

#### EMG processing.

EMG data were first decimated (data were low-pass filtered using a Chebyshev Type I filter at 200 Hz before downsampling) to 500 Hz and subsequently high-pass filtered at 20 Hz (4th order Butterworth filter), motivated by the fact that the power density function of surface EMG signals has insignificant contributions at frequencies <10 Hz (Merletti and Di Torino 1999). Data were subsequently divided into respective movement periods (ECC, ISO, CON). We estimated power spectral densities (PSD) according to Welch’s method. To investigate EMG amplitude impact on IMC, we estimated mean EMG amplitudes by way of calculating root mean square (RMS) values across 50-ms windows for all muscles and periods. Two one-way repeated measures ANOVA (rmANOVA) were conducted for factors PERIOD and MUSCLE to determine differences in mean EMG amplitude for all muscles and movement periods, with post hoc Bonferroni-Holm tests being carried out when appropriate. For this purpose, EMG activities were normalized to maximum values measured across the entire recording for each muscle, respectively (i.e., activation ratio) (Pizzamiglio et al. 2017). This was done to minimize variance across subjects due to potential variations in electrode placements and skin impedances (Pizzamiglio et al. 2017). For all statistical comparisons, a *P* value of *P* < 0.05 was considered significant. All *P* values adjusted for multiple comparisons are reported with the results.

#### EMG signal analysis.

IMC analysis was carried out calculating coherence between all possible EMG-EMG combinations of homologous muscle pairs. Band-pass filtered EMG data were rectified using the Hilbert transform (Boonstra et al. 2015). This procedure extracts EMG-signal envelopes and provides similar results compared with standard full-wave rectification (Boonstra and Breakspear 2012; Boonstra et al. 2015). Data were epoched per movement period, yielding 40 trials per period, which were concatenated in a final step. Intermuscular coherence and cross-power spectral density (CPSD) were estimated between pairs of concatenated EMG data using Welch’s method with a Hanning window of 500 ms and an overlap of 75% (Boonstra et al. 2015; Pizzamiglio et al. 2017):

$$\left|C_{xy}(f)\right|=\frac{{\left|S_{xy}(f)\right|}^{2}}{S_{xx}(f)S_{yy}(f)}$$

where *S_xy_*(*f*) is the CPSD and *S_xx_*(*f*) and *S_yy_*(*f*) represent the PSD of both input signals *x*(*t*) and *y*(*t*), i.e., any pairwise combination of the investigated muscles, respectively (Mima and Hallett 1999; Rosenberg et al. 1989). To evaluate the significance of IMC results, confidence limits (α = 5%; *P* < 0.05) were calculated according to Rosenberg et al. (1989):

$$CL_{\alpha }=1-{\left(1-\frac{\alpha }{100}\right)}^{\frac{1}{N-1}}$$

where *N* is the number of disjoint segments and CL reflects the confidence limit above which observed coherence values are considered significant. Confidence limits were subsequently adjusted to account for overlapping segments (Terry and Griffin 2008; Welch 1967). Phase lags between homologous muscle pairs were estimated by calculating phase angles φ(*f*) from complex valued CPSD (Rosenberg et al. 1989):

$$\varphi \left(f\right)=tan^{-1}[S_{xy}\left(f\right)]$$

Furthermore, we calculated the slope of the phase angle per frequency band of interest and subsequently multiplied each slope by 1/2π to identify temporal delays between homologous muscle pairs during all movement periods (Grosse et al. 2002).

For statistical analyses, significant IMC estimates were summed across three frequency bands of interest: *1*) alpha (8–12 Hz), *2*) beta (13–30 Hz), and *3*) gamma (30–44 Hz) (Laine and Valero-Cuevas 2017). IMC was analyzed as areas of coherence, i.e., summed IMC estimates (*IMC*_area_) over specific frequency bands rather than peak coherence. Analyzing areas of coherence estimates has been deemed superior compared with analyzing peak values and frequencies of coherence estimates (Jaiser et al. 2016; Omlor et al. 2007; Spedden et al. 2019; Ushiyama et al. 2010). *IMC*_area_ were then pooled for homologous muscles and movement periods. Two-way rmANOVAs were conducted to determine frequency band-specific differences in *IMC*_area_ between homologous muscles and movement periods, with post hoc Bonferroni-Holm tests being carried out when appropriate. To avoid skewness and normalize variance, all data were log-transformed before statistical analyses. Differences in temporal delays were also analyzed by way of two-way rmANOVA per homologous muscle pair and movement period for each frequency band of interest with post hoc Bonferroni-Holm tests being carried out when appropriate. For all statistical comparisons, a *P* value of *P* < 0.05 was considered significant. All *P* values adjusted for multiple comparisons are reported with the results. Please note that statistical analyses of *IMC*_area_ and temporal delays were performed only between homologous muscle pairs. Therefore, muscles of interest are listed as VL (regarding estimates between VL right and VL left), VM, TA, and ES, throughout the results section.

#### Data accessibility.

The data that support the findings of this study are available on request from the corresponding author, R. Kenville. The data are not publicly available due to data protection policies practiced at our institute (Max Planck Institute for cognitive and brain sciences in Leipzig), e.g., their containing information that could compromise the privacy of research participants.

## RESULTS

**Fig. 1.**
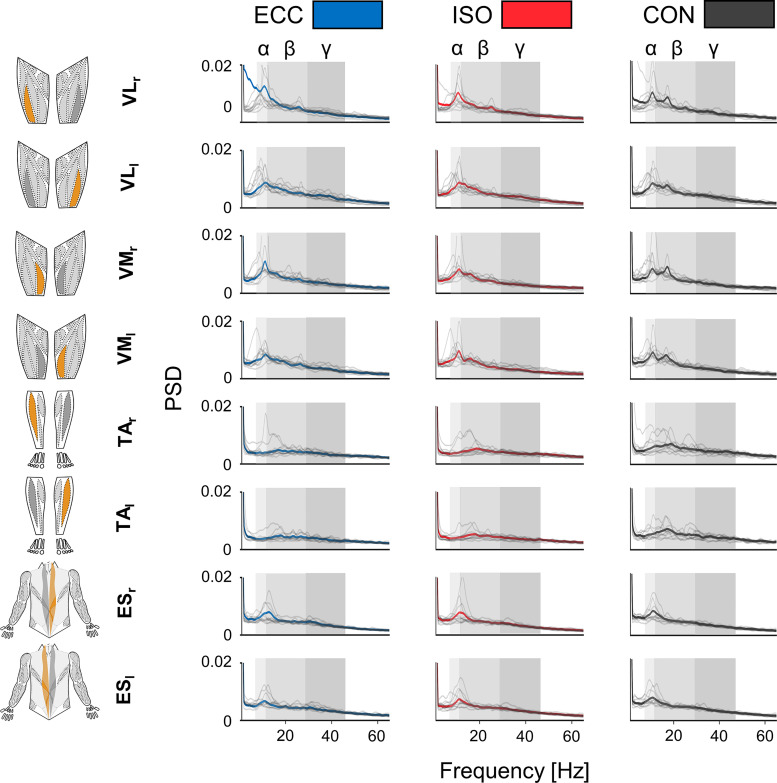
Normalized power spectral density (PSD) of electromyography (EMG) envelopes per muscle and period. PSD of EMG envelopes are illustrated for all muscles during each movement period. Power spectra were averaged across muscles, epochs, and participants and normalized to total power. Mean values of normalized PSD are displayed per movement period: eccentric movement period (ECC; blue), isometric movement period (ISO; red), and concentric movement period (CON; gray), while individual values are displayed as gray lines. Each row represents distinct muscles which are highlighted in orange. Alpha, beta, and gamma frequency bands are indicated through rectangles colored in different gradations of gray. ES_l_ and ES_r_, left and right erector spinae; TA_l_ and TA_r_, left and right tibialis anterior; VL_l_ and VL_r_, left and right vastus lateralis; VM_l_ and VM_r_, left and right vastus medialis.

As an introductory overview, Fig. 1 illustrates average power spectral densities (PSD) of all muscles during different periods of BpS. We first inspected spectral contents of all EMG envelopes by assessing normalized PSD. PSD revealed broad spectra maximum around 10 Hz for all muscles and conditions (cf. Fig. 1). For both VL and VM, a broad spectrum with a peak around 20 Hz was also visible.

### 

#### Intermuscular coherence.

**Fig. 2.**
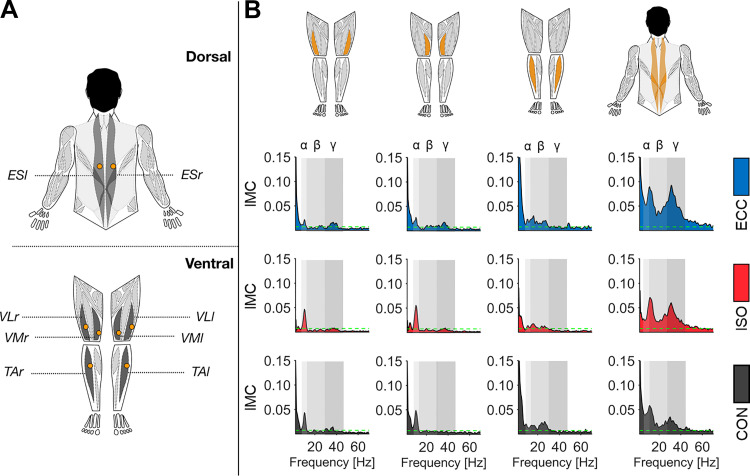
Overview of intermuscular coherence (IMC) spectra I. *A*: schematic setup of electromyography (EMG) sensors. All EMG recording sites are highlighted through blacked-out muscles with respective labels situated beside them. Please note that the upper half is drawn from dorsal perspective and the lower half is drawn from ventral perspective. Orange circles indicate EMG sensor positions. *B*: grand-averaged IMC for all opposing muscle pairs (highlighted in orange). Each row of IMC spectra represents different movement periods: eccentric movement period (ECC; blue), isometric movement period (ISO; red), and concentric movement period (CON; gray). Dashed green lines indicate confidence limit (CL) above which observed coherence values are considered significant. Alpha, beta, and gamma frequency bands are indicated through rectangles colored in different gradations of gray. ES_l_ and ES_r_, left and right erector spinae; TA_l_ and TA_r_, left and right tibialis anterior; VL_l_ and VL_r_, left and right vastus lateralis; VM_l_ and VM_r_, left and right vastus medialis.

A two-way rmANOVA (factors: MUSCLE and PERIOD) was carried out for log-transformed *IMC*_area_ in each frequency band of interest. Please see Fig. 2*B* for an overview regarding differences in *IMC*_area_ between movement periods.

**Fig. 3.**
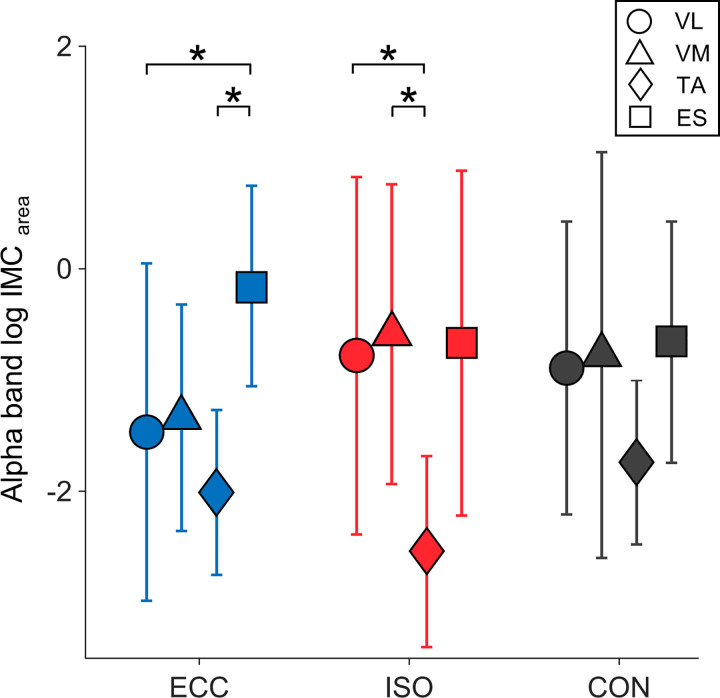
Averaged alpha *IMC*_area_ per muscle and movement period. Averaged log-transformed *IMC*_area_ are illustrated per muscle and period for alpha frequency band. *Significant differences between *IMC*_area_ of different muscles within movement periods. Respective *P* values are reported in the results section. Here, blue symbols indicate *IMC*_area_ for eccentric movement period (ECC), whereas red symbols indicate *IMC*_area_ for isometric movement period (ISO) and black symbols represent *IMC*_area_ for concentric movement period (CON). Here, circles indicate *IMC*_area_ for vastus lateralis (VL), triangles for vastus medialis (VM), diamonds for tibialis anterior (TA), and squares for erector spinae (ES). *IMC*_area_, summed intermuscular coherence estimates.

**Fig. 4.**
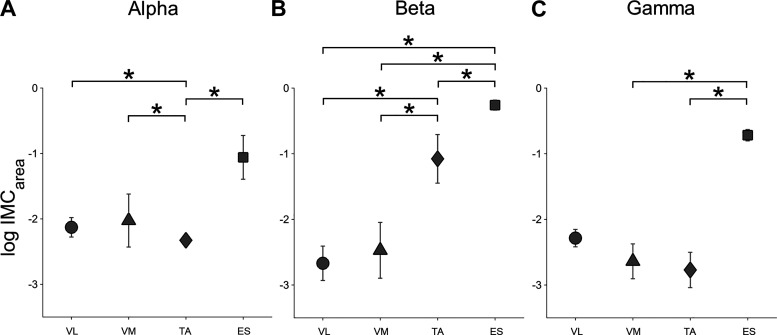
Averaged *IMC*_area_ per muscle. Averaged log-transformed *IMC*_area_ are illustrated per muscle for alpha (*A*), beta (*B*), and gamma (*C*) frequency bands. *Significant differences between *IMC*_area_ of different muscles. Respective *P* values are reported in the results section. Here, circles indicate *IMC*_area_ vastus lateralis (VL), triangles for vastus medialis (VM), diamonds for tibialis anterior (TA), and squares for erector spinae (ES). *IMC*_area_, summed intermuscular coherence estimates.

We found a significant interaction between MUSCLE*PERIOD for alpha *IMC*_area_ [*P* = 0.004; cf. Supplemental Table S1 (all Supplemental tables are available at https://doi.org/10.6084/m9.figshare.12618992)], with post hoc Bonferroni-Holm tests revealing *IMC*_area_ to be higher during ECC for ES versus TA and VL and higher during ISO for ES versus TA, as well as lower for TA versus VL and VM (cf. Fig. 3). Additionally, we found a main effect for MUSCLE (*P* = 2.867 × 10^−4^, cf. Supplemental Table S1) with post hoc Bonferroni-Holm tests revealing *IMC*_area_ to be higher for ES versus TA, as well as lower for TA versus VL and VM (cf. Fig. 4*A*). For a detailed overview relating to post hoc results of alpha *IMC*_area_, please see Supplemental Table S5.

**Fig. 5.**
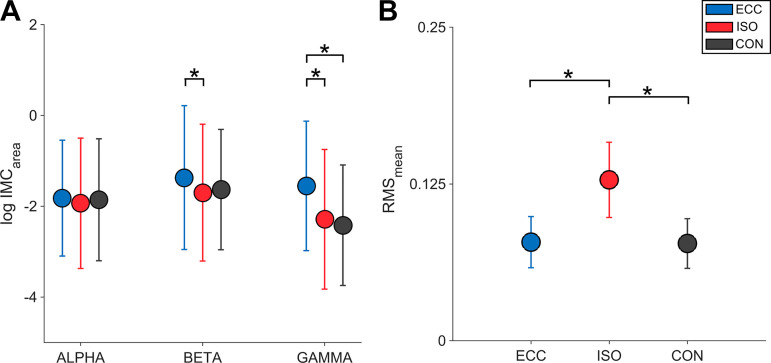
Averaged *IMC*_area_ and mean EMG amplitudes (RMS) per movement period. *A*: averaged log-transformed *IMC*_area_ are illustrated per period for alpha, beta, and gamma frequency bands. *B*: averaged, normalized root mean square (RMS_mean_) values are depicted per period. *Significant differences between *IMC*_area_ of different movement periods. Respective *P* values are reported in the results section. Here, blue symbols indicate *IMC*_area_ for eccentric movement period (ECC), whereas red symbols indicate *IMC*_area_ for isometric movement period (ISO) and black symbols represent *IMC*_area_ for concentric movement period (CON). EMG, electromyography; *IMC*_area_, summed intermuscular coherence estimates.

Regarding beta *IMC*_area_ we found a significant interaction between MUSCLE*PERIOD (*P* = 0.001, cf. Supplemental Table S2), although post hoc tests failed to reach significance. We also found a main effect for MUSCLE (*P* = 2.260 × 10^−6^, cf. Supplemental Table S2) with post hoc Bonferroni-Holm tests revealing *IMC*_area_ to be higher for ES versus TA, VL, and VM, as well as higher for TA versus VL and VM (cf. Fig. 4*B*). Furthermore, a main effect for PERIOD was found (*P* = 0.032, cf. Supplemental Table S2) with post hoc Bonferroni-Holm tests revealing *IMC*_area_ to be higher for ECC versus ISO (cf. Fig. 5*A*). For a detailed overview relating to post hoc results of beta *IMC*_area_ please see Supplemental Table S6.

For gamma *IMC*_area_ we found main effects for MUSCLE (*P* = 0.004, cf. Supplemental Table S3), with post hoc Bonferroni-Holm tests revealing *IMC*_area_ to be higher for ES versus TA and VM (cf. Fig. 4*C*) and PERIOD (*P* = 0.019, cf. Supplemental Table S3) with post hoc Bonferroni-Holm tests revealing *IMC*_area_ to be higher for ECC versus ISO and lower for CON versus ECC (cf. Fig. 5*A*). For a detailed overview relating to post hoc results of gamma *IMC*_area_ please see Supplemental Table S7.

To provide an overview of the common input of all muscle pairs despite the focus of this study on homologous muscle pairs, a complete presentation of all IMC spectra is given in Supplemental Figure S2.

#### Phase angle and temporal delay.

**Fig. 6.**
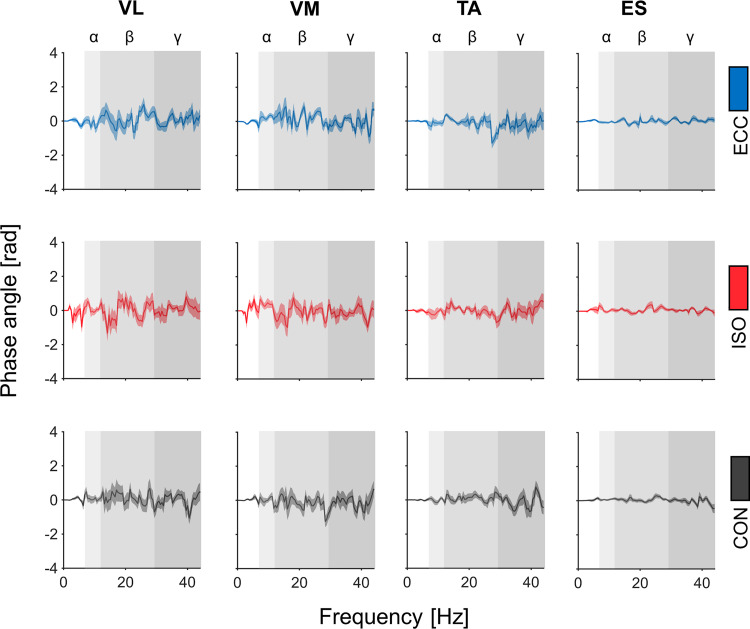
Overview of phase angle spectra. Each column depicts averaged phase angle spectra and corresponding standard errors of the mean for all opposing muscle pairs. Each row represents phase angle spectra of different movement periods: eccentric movement period (ECC; blue), isometric movement period (ISO; red), and concentric movement period (CON; gray). Alpha, beta, and gamma frequency bands are indicated through rectangles colored in different gradations of gray.

A detailed overview of mean phase angle spectra across homologous muscle pairs and movement periods is provided in Fig. 6. One-sample *t* tests of phase angles did not reveal significant differences from zero per muscle or movement period across frequency bands.

Additionally, statistical analyses of temporal delays across all frequency bands of interest did not reveal any significant effects for MUSCLE (alpha: *P* = 0.167; beta: *P* = 0.235; gamma: *P* = 0.702), PERIOD (alpha: *P* = 0.357; beta: *P* = 0.413; gamma: *P* = 0.638) or a MUSCLE*PERIOD interaction (alpha: *P* = 0.592; beta: *P* = 0.101; gamma: *P* = 0.956).

#### EMG amplitudes.

Mean EMG amplitude comparisons revealed main effects for MUSCLE (*P* = 1.691 × 10^−4^, cf. Supplemental Table S4) and PERIOD (*P* = 4.743 × 10^−12^, cf. Supplemental Table S4). Mean EMG amplitudes were significantly higher for ISO compared with CON and ECC, whereas no statistically significant differences could be observed between CON and ECC (cf. Fig. 5*B*). Post hoc Bonferroni-Holm tests revealed significant mean EMG amplitude differences between both ES and TA, as well as TA and VM. For a detailed overview relating to post hoc results of mean EMG amplitudes please see Supplemental Table S8.

## DISCUSSION

In the present study, we investigated intermuscular interactions between principal homologous muscles involved in BpS during dynamic (ECC and CON) and static (ISO) movement periods to uncover bilateral aspects of compound motor control. Consistent with our hypotheses, we found significant IMC in all frequency bands of interest (alpha, beta, and gamma) across all homologous muscle pairs and movement periods. Statistical analyses revealed movement period-related modulations of beta and gamma IMC (both highest during ECC), as well as differences in the magnitude of IMC between pairs of principal homologous muscles across alpha, beta, and gamma frequency bands. Taken together, our findings demonstrate complex central nervous processing of homologous muscle control during BpS. Specifically, our evidence contributes to the understanding of compound motor control, as for the first time, we reveal movement-period related modulations of central nervous processing in the control of homologous muscles during a compound movement. All findings are discussed in detail in the following.

### 

#### Intermuscular coherence during BpS.

Intermuscular synchronization is an important determinant of successful motor execution. Accordingly, there have been indications that the central nervous system alters intermuscular synchronization as a function of movement demands (Clark et al. 2013; van Asseldonk et al. 2014). Previous research on IMC during squat movements uncovered IMC between upper thigh muscles (VL and VM) in frequency ranges between 15 and 80 Hz (Mohr et al. 2015, 2018). Here, we also observed IMC in alpha, beta, and gamma frequency bands across participants, muscles, and periods.

#### Alpha band IMC.

Our findings show alpha band IMC between all homologous muscle pairs during BpS. Here, greatest IMC was found in ES followed by VM, VL, and TA. This is in line with numerous studies that also observed alpha band IMC in ES (Danna-Dos-Santos et al. 2014; Degani et al. 2017), VM (Boonstra et al. 2008, 2015), and TA (Boonstra et al. 2009, 2015; Bravo-Esteban et al. 2014). Out of these studies, three analyzed coherence between homologous muscles (VM and TA) (Boonstra et al. 2008, 2009, 2015), whereas two other studies examined IMC between ES and biceps femoris (Danna-Dos-Santos et al. 2014; Degani et al. 2017). It should be noted that in both studies EMG electrodes for ES recordings were placed largely around the lumbar region of the spine compared with thoracic placement in the present study. Functionally, alpha IMC in ES could reflect postural stability maintenance, as it was observed previously in a bipedal stance task (Danna-Dos-Santos et al. 2014). Furthermore, earlier studies showed TA to exhibit IMC at low frequency ranges around 10 Hz during upright stance (Danna-Dos-Santos et al. 2014, 2015), potentially reflecting a strategy to monitor degrees of freedom and also providing stability during such a movement. Additionally, the speed of BpS execution, which was comparably low (15 s for one repetition) in the present study could be a reason for this finding, as alpha band IMC has been observed in slow movements before (Kouzaki and Masani 2012). Furthermore, we were unable to observe significant differences in alpha IMC between movement periods. This finding is in line with previous research showing alpha IMC to be stable across concentric and eccentric contractions in upper extremities (Nguyen et al. 2017) and now we extend these conclusions for lower extremities as well. Still, as this is the first study examining differences in alpha IMC between homologous muscles during BpS, more evidence is needed to draw definite conclusions. Neurophysiological origins of alpha IMC are thought to relate to subcortical and spinal structures (Boonstra et al. 2009; Grosse and Brown 2003; Laine and Valero-Cuevas 2017). It is assumed that common input at alpha frequencies is reflective of spinal interneuron activity connecting and integrating sensory afferents of functionally bound muscles. Although this concept needs to be demonstrated in humans, it is well described in animal studies (Hart and Giszter 2010; Kargo and Giszter 2000; Levine et al. 2014). Therefore, it seems that alpha IMC, as observed in this study, could be reflective of subcortical and or spinal maintenance processing.

#### Beta band IMC.

We found beta IMC between all homologous muscle pairs recorded during BpS. Here, most pronounced IMC was again evident in ES followed by TA, VM, and VL, as both ES and TA showed increases in IMC compared with VM and VL. Our findings add to previous evidence of beta IMC for ES (Danna-Dos-Santos et al. 2014), TA (Boonstra et al. 2008, 2009, 2015; Bravo-Esteban et al. 2014), VM (Boonstra et al. 2008; von Tscharner et al. 2018; Walker et al. 2019), and VL (von Tscharner et al. 2018; Walker et al. 2019), but were expected, as beta IMC has been commonly observed between synergistically activated muscles (Boonstra 2013; Boonstra and Breakspear 2012; Castronovo et al. 2018; Degani et al. 2017; Kilner et al. 1999). Beta IMC of the lower extremities has been described between homologous VM during bilateral leg extensions (Boonstra et al. 2008), as well as homologous VM and TA during standing (Boonstra et al. 2015), although not for homologous ES. The observed differences in beta IMC between muscles within our study may reflect differences in muscle functioning within BpS. Both ES and TA only slightly change their length during BpS execution, whereby their main function is the maintenance of posture (Lee et al. 2016).

Additionally, ES motor control during BpS most likely underlies an established neural blueprint, since ES functions during BpS and daily human routines, i.e., maintaining posture, are comparable. A possible explanation for the observed differences in IMC between muscles refers back to the main function of both ES and TA, which collectively enable upper and lower body stability throughout BpS (Myer et al. 2014). It was demonstrated during specific motor tasks that more strongly coordinated homologous muscles showed increased IMC magnitudes (de Vries et al. 2016; Kisiel-Sajewicz et al. 2011). Therefore, both homologous ES and TA may participate in the maintenance of posture whereas VM and VL function as primary sources of muscular force production throughout BpS performance. Supporting evidence is provided by studies showing EMG activity of VM to increase as a function of load compared with ES during BpS (Yavuz and Erdag 2017).

With this study, we also show beta IMC to be higher during ECC as compared with ISO. This was unexpected as we hypothesized beta IMC to be highest during ISO. Previous studies observed beta IMC to be more pronounced or present in general during static movements (ISO) compared with dynamic movements (ECC and CON) (Baker 2007; Kilner et al. 1999; Reyes et al. 2017). There is evidence for a decrease in beta IMC between static and dynamic movements (Kilner et al. 1999), although contrasting results also exist (Laine and Valero-Cuevas 2017). Some authors clarify that this decrease in beta IMC only applies to dynamic movements that rely on highly individuated control of all involved muscles (Laine and Valero-Cuevas 2017; Reyes et al. 2017). Accordingly, it was shown that separate successive finger movements reduced beta IMC between finger muscles (Reyes et al. 2017). In this context, our results indirectly support these findings, as BpS motor control is achieved through common input stemming from corticospinal projections to functionally relevant musculature (Mohr et al. 2015) and likely requires a more synergistic control of involved muscles, as is evident from other compound motor tasks such as standing (Boonstra et al. 2015) and cycling (De Marchis et al. 2015). Regarding the underlying neuronal source of beta IMC, it is thought that IMC in this frequency range presumably reflects a coordinated neuronal drive to functionally connected muscles originating from the motor cortex (Reyes et al. 2017). Several studies support the view that neurons of the pyramidal tracts are primarily responsible for the generation of beta oscillations (Baker et al. 2003; Jackson et al. 2002; Roopun et al. 2006). Furthermore, beta IMC does not appear to be strongly influenced by somatosensory feedback, but rather by the type of movement (Nguyen et al. 2017). Bilateral movement coordination through beta IMC is therefore regarded as a corticofugal mechanism for efficient control of synergistic and thus bilateral movement control (Nguyen et al. 2017). It therefore seems reasonable that all muscles we recorded during BpS collectively act as prime movers of that movement and thus are likely to reflect a common central neuronal control (De Luca and Erim 2002) as opposed to individuated control.

#### Gamma band IMC.

Our results show gamma IMC in all examined muscles with significant differences between ES and TA as well as ES and VL. Apart from ES, gamma IMC has been observed in all other muscles recorded during this study, i.e., VM, VL (Mohr et al. 2015, 2018; von Tscharner et al. 2018), and TA (Bravo-Esteban et al. 2014; van Asseldonk et al. 2014). Numerous studies have observed gamma IMC between muscle pairs of lower extremities (Bravo-Esteban et al. 2014; Castronovo et al. 2018; De Marchis et al. 2015; Mohr et al. 2015, 2018; van Asseldonk et al. 2014; Walker et al. 2019), although, to the best of our knowledge, this is the first study to demonstrate gamma IMC between homologous muscles of lower extremities. With these results, we extend findings by Mohr and colleagues (Mohr et al. 2015, 2018), who originally observed gamma IMC between VM and VL during isometric as well as dynamic BpS. In addition to differences between muscles, we observed significant differences in gamma IMC between ECC versus ISO, as well as ECC versus CON. In line with our hypotheses, gamma IMC was greatest during ECC compared with both ISO and CON. Previous research suggested that gamma IMC is likely to reflect sensory integration (De Marchis et al. 2015; Nguyen et al. 2017). This is reinforced by the fact that most tasks requiring strenuous processing and integration due to task complexity, high force demands, or novelty of respective movements show strong gamma IMC (De Marchis et al. 2015; Mohr et al. 2015, 2018; von Tscharner et al. 2018). Furthermore, it is known that proprioceptive feedback of muscle spindles increases during lengthening/eccentric (ECC) contractions (Burke et al. 1978; Duchateau and Enoka 2008). This, in turn, would indeed explain why gamma IMC was particularly strong during the eccentric part of the squat. Regarding intermuscular synchronization, IMC was shown to increase when comparing dynamic and isometric contractions of upper and lower extremities (Semmler et al. 2002; von Tscharner 2014). Taken together, it seems plausible that the increase observed between ECC and ISO/CON is related to an increase in sensory information integration due to various BpS properties such as complexity and/or novelty of the movement. This may indicate that gamma IMC is increased where proprioception is particularly necessary due to task- and movement period-specific demands on the muscles.

#### Critical perspective.

Although IMC is an established research tool in humans, there are several factors potentially influencing its detection (Semmler et al. 2013). For example, IMC results generally show high inter- and intraindividual variability (Jaiser et al. 2016). This could be due to anatomical specificity of muscles and their innervation as well as individual patterns of motor control. Electrode positioning is also critical in assessing IMC (Keenan et al. 2012). Concerning this matter, we were able to maintain relative electrode positions across participants by following standard SENIAM EMG electrode position guidelines (Hermens et al. 2000), rendering this issue negligible. A more broadband debate revolves around the rectification of EMG signals. This issue has been ongoing for over a decade with numerous studies providing evidence regarding rectification effects on EMG signals (Farina et al. 2004; Myers et al. 2003; Yao et al. 2007). In short, empirical studies promote EMG rectification (Boonstra et al. 2008; Mima and Hallett 1999; Yao et al. 2007), as opposed to simulation studies that largely argue against rectification (Neto and Christou 2010; Stegeman et al. 2010). Here, we used rectification as it is thought to better reflect the information about the firing rate of motor units within EMG signals (Semmler et al. 2013) and it increases the comparability of our results as most other IMC studies have used rectification within EMG preprocessing (Boonstra et al. 2008, 2009; Danna-Dos-Santos et al. 2010, 2014; Keenan et al. 2012; Laine and Valero-Cuevas 2017; Poston et al. 2010). Another important factor is the potential impact of EMG signal amplitudes on intermuscular coherence (Bayraktaroglu et al. 2013; Singh and Prakash 2000). To address this issue, we computed and compared mean EMG amplitudes between muscles and movement periods to examine similarities in the observed effects between EMG amplitudes and IMC. Mean EMG amplitudes were highest during ISO compared with ECC and CON throughout all muscles and participants (cf. Fig. 5*B*), whereas IMC showed differential modulations across movement periods, homologous muscles, and participants (cf. Fig. 5*A*). Therefore, it seems unlikely that the changes in EMG amplitudes were primarily responsible for our IMC results as in this case one would expect a close relationship between the increase in EMG amplitudes and the strength of IMC, which was not the case in our study. Lastly, EMG-EMG cross talk possibly confounds IMC measures. However, this issue is unlikely to explain our results since we computed coherence between homologous muscles that were located on opposing limbs, thus effectively eliminating the leakage of the EMG signals between corresponding recording electrodes. A notable exception are both ES muscles which were spaced apart by roughly 7 cm, although it seems unreasonable that the alleged cross talk should only affect certain frequency ranges (Clark et al. 2013).

#### Conclusion.

In summary, we provide novel evidence that, during BpS, homologous muscle functions are mediated through common oscillatory inputs spanning across alpha, beta, and gamma frequencies with distinct synchronization patterns at different movement periods. We show that for beta and gamma IMC the magnitude of common input is greater in dynamic movement periods (ECC and CON) when compared with static periods (ISO). We also show that homologous muscle pairs involved in postural (ES) and bipedal (TA) stability maintenance, exhibit greater IMC compared with those involved in primary force production during BpS (VM and VL). In general, these findings reflect task-dependent central nervous processing of synchrony between homologous muscles through magnitude and frequency modulations. Furthermore, we suggest that the observation of significant IMC in different frequency bands is reflective of modulatory distinctions between spinal/subcortical involvement (alpha), a functional divergence between muscle groups (beta), and increased sensory information processing (gamma) that together achieve appropriate intermuscular control during BpS. With this study, we extend previous knowledge by uncovering movement period-related modulations in central nervous processing in homologous muscles during a compound movement. This evidence may facilitate the application of IMC during compound movements in the areas of athletic performance and rehabilitation.

## GRANTS

This study was supported by the Max-Planck Society. V.V.N. was supported in part by the HSE Basic Research Program and the Russian Academic Excellence Project ‘5–100.’

## DISCLOSURES

No conflicts of interest, financial or otherwise, are declared by the authors.

## AUTHOR CONTRIBUTIONS

R.K. and V.V.N. conceived and designed the research; R.K., T.M. and D.M. performed experiments; R.K., T.M., C.V., D.M. and V.V.N. analyzed data; R.K., T.M., C.V. and V.V.N. interpreted results of experiments; D.M. prepared figures; R.K. drafted manuscript; R.K., T.M., C.V., D.M., A.V., P.R. and V.V.N. edited and revised manuscript; R.K., T.M., C.V., D.M., A.V., P.R. and V.V.N. approved final version of manuscript.

## References

[B1] Aune TK, Aune MA, Ettema G, Vereijken B Comparison of bilateral force deficit in proximal and distal joints in upper extremities. Hum Mov Sci 32: 436–444, 2013. doi:10.1016/j.humov.2013.01.005. 23719626

[B2] Baker SN Oscillatory interactions between sensorimotor cortex and the periphery. Curr Opin Neurobiol 17: 649–655, 2007. doi:10.1016/j.conb.2008.01.007. 18339546 PMC2428102

[B3] Baker SN, Kilner JM, Pinches EM, Lemon RN The role of synchrony and oscillations in the motor output. Exp Brain Res 128: 109–117, 1999. doi:10.1007/s002210050825. 10473748

[B4] Baker SN, Pinches EM, Lemon RN Synchronization in monkey motor cortex during a precision grip task. II. effect of oscillatory activity on corticospinal output. J Neurophysiol 89: 1941–1953, 2003. doi:10.1152/jn.00832.2002. 12686573

[B5] Bayraktaroglu Z, von Carlowitz-Ghori K, Curio G, Nikulin VV It is not all about phase: amplitude dynamics in corticomuscular interactions. Neuroimage 64: 496–504, 2013. doi:10.1016/j.neuroimage.2012.08.069. 22960151

[B6] Boonstra TW The potential of corticomuscular and intermuscular coherence for research on human motor control. Front Hum Neurosci 7: 855, 2013. doi:10.3389/fnhum.2013.00855. 24339813 PMC3857603

[B7] Boonstra TW, Breakspear M Neural mechanisms of intermuscular coherence: implications for the rectification of surface electromyography. J Neurophysiol 107: 796–807, 2012. doi:10.1152/jn.00066.2011. 22072508

[B8] Boonstra TW, Daffertshofer A, Roerdink M, Flipse I, Groenewoud K, Beek PJ Bilateral motor unit synchronization of leg muscles during a simple dynamic balance task. Eur J Neurosci 29: 613–622, 2009. doi:10.1111/j.1460-9568.2008.06584.x. 19175407

[B9] Boonstra TW, Daffertshofer A, van Ditshuizen JC, van den Heuvel MR, Hofman C, Willigenburg NW, Beek PJ Fatigue-related changes in motor-unit synchronization of quadriceps muscles within and across legs. J Electromyogr Kinesiol 18: 717–731, 2008. doi:10.1016/j.jelekin.2007.03.005. 17462912

[B10] Boonstra TW, Danna-Dos-Santos A, Xie HB, Roerdink M, Stins JF, Breakspear M Muscle networks: connectivity analysis of EMG activity during postural control. Sci Rep 5: 17830, 2015. doi:10.1038/srep17830. 26634293 PMC4669476

[B11] Bravo-Esteban E, Taylor J, Aleixandre M, Simon-Martínez C, Torricelli D, Pons JL, Gómez-Soriano J Tibialis Anterior muscle coherence during controlled voluntary activation in patients with spinal cord injury: diagnostic potential for muscle strength, gait and spasticity. J Neuroeng Rehabil 11: 23, 2014. doi:10.1186/1743-0003-11-23. 24594207 PMC3973993

[B12] Brown P, Farmer SF, Halliday DM, Marsden J, Rosenberg JR Coherent cortical and muscle discharge in cortical myoclonus. Brain 122: 461–472, 1999. doi:10.1093/brain/122.3.461. 10094255

[B13] Buckthorpe MW, Pain MT, Folland JP Bilateral deficit in explosive force production is not caused by changes in agonist neural drive. PLoS One 8: e57549, 2013. doi:10.1371/journal.pone.0057549. 23472091 PMC3589403

[B14] Burke D, Hagbarth KE, Löfstedt L Muscle spindle activity in man during shortening and lengthening contractions. J Physiol 277: 131–142, 1978. doi:10.1113/jphysiol.1978.sp012265. 148511 PMC1282382

[B15] Casale P, Pujol O, Radeva P Human activity recognition from accelerometer data using a wearable device In: Pattern Recognition and Image Analysis. IbPRIA 2011, edited by Vitrià J, Sanches JM, Hernández M Berlin: Springer, 2011, p. 289–296. Lecture Notes in Computer Science 6669. doi:10.1007/978-3-642-21257-4_36.

[B16] Castronovo AM, De Marchis C, Schmid M, Conforto S, Severini G Effect of task failure on intermuscular coherence measures in synergistic muscles. Appl Bionics Biomech 2018: 4759232, 2018. doi:10.1155/2018/4759232. 29967654 PMC6008706

[B17] Cauraugh JH, Lodha N, Naik SK, Summers JJ Bilateral movement training and stroke motor recovery progress: a structured review and meta-analysis. Hum Mov Sci 29: 853–870, 2010. doi:10.1016/j.humov.2009.09.004. 19926154 PMC2889142

[B18] Cauraugh JH, Summers JJ Neural plasticity and bilateral movements: a rehabilitation approach for chronic stroke. Prog Neurobiol 75: 309–320, 2005. doi:10.1016/j.pneurobio.2005.04.001. 15885874

[B19] Chang YJ, Chou CC, Chan HL, Hsu MJ, Yeh MY, Fang CY, Chuang YF, Wei SH, Lien HY Increases of quadriceps inter-muscular cross-correlation and coherence during exhausting stepping exercise. Sensors (Basel) 12: 16353–16367, 2012. doi:10.3390/s121216353. 23443382 PMC3571786

[B20] Clark DJ, Kautz SA, Bauer AR, Chen YT, Christou EA Synchronous EMG activity in the piper frequency band reveals the corticospinal demand of walking tasks. Ann Biomed Eng 41: 1778–1786, 2013. doi:10.1007/s10439-013-0832-4. 23740367 PMC3725573

[B21] Danna-Dos-Santos A, Boonstra TW, Degani AM, Cardoso VS, Magalhaes AT, Mochizuki L, Leonard CT Multi-muscle control during bipedal stance: an EMG-EMG analysis approach. Exp Brain Res 232: 75–87, 2014. doi:10.1007/s00221-013-3721-z. 24105595

[B22] Danna-Dos-Santos A, Degani AM, Boonstra TW, Mochizuki L, Harney AM, Schmeckpeper MM, Tabor LC, Leonard CT The influence of visual information on multi-muscle control during quiet stance: a spectral analysis approach. Exp Brain Res 233: 657–669, 2015. doi:10.1007/s00221-014-4145-0. 25407521

[B23] Danna-Dos Santos A, Poston B, Jesunathadas M, Bobich LR, Hamm TM, Santello M Influence of fatigue on hand muscle coordination and EMG-EMG coherence during three-digit grasping. J Neurophysiol 104: 3576–3587, 2010. doi:10.1152/jn.00583.2010. 20926609 PMC3007653

[B24] De Luca CJ, Erim Z Common drive in motor units of a synergistic muscle pair. J Neurophysiol 87: 2200–2204, 2002. doi:10.1152/jn.00793.2001. 11929938

[B25] De Marchis C, Severini G, Castronovo AM, Schmid M, Conforto S Intermuscular coherence contributions in synergistic muscles during pedaling. Exp Brain Res 233: 1907–1919, 2015. doi:10.1007/s00221-015-4262-4. 25821181

[B26] de Vries IE, Daffertshofer A, Stegeman DF, Boonstra TW Functional connectivity in the neuromuscular system underlying bimanual coordination. J Neurophysiol 116: 2576–2585, 2016. doi:10.1152/jn.00460.2016. 27628205 PMC5133293

[B27] Degani AM, Leonard CT, Danna-Dos-Santos A The use of intermuscular coherence analysis as a novel approach to detect age-related changes on postural muscle synergy. Neurosci Lett 656: 108–113, 2017. doi:10.1016/j.neulet.2017.07.032. 28732761

[B28] Dideriksen JL, Negro F, Falla D, Kristensen SR, Mrachacz-Kersting N, Farina D Coherence of the surface EMG and common synaptic input to motor neurons. Front Hum Neurosci 12: 207, 2018. doi:10.3389/fnhum.2018.00207. 29942254 PMC6004394

[B29] Duchateau J, Enoka RM Neural control of shortening and lengthening contractions: influence of task constraints. J Physiol 586: 5853–5864, 2008. doi:10.1113/jphysiol.2008.160747. 18955381 PMC2655422

[B30] Farina D, Merletti R, Enoka RM The extraction of neural strategies from the surface EMG. J Appl Physiol (1985) 96: 1486–1495, 2004. doi:10.1152/japplphysiol.01070.2003. 15016793

[B31] Farmer SF Rhythmicity, synchronization and binding in human and primate motor systems. J Physiol 509: 3–14, 1998. doi:10.1111/j.1469-7793.1998.003bo.x. 9547376 PMC2230956

[B32] Grabowska A Sex on the brain: are gender-dependent structural and functional differences associated with behavior? J Neurosci Res 95: 200–212, 2017. doi:10.1002/jnr.23953. 27870447

[B33] Graci V, Van Dillen LR, Salsich GB Gender differences in trunk, pelvis and lower limb kinematics during a single leg squat. Gait Posture 36: 461–466, 2012. doi:10.1016/j.gaitpost.2012.04.006. 22591790 PMC3407338

[B34] Gross J, Timmermann L, Kujala J, Dirks M, Schmitz F, Salmelin R, Schnitzler A The neural basis of intermittent motor control in humans. Proc Natl Acad Sci USA 99: 2299–2302, 2002. doi:10.1073/pnas.032682099. 11854526 PMC122359

[B35] Grosse P, Brown P Acoustic startle evokes bilaterally synchronous oscillatory EMG activity in the healthy human. J Neurophysiol 90: 1654–1661, 2003. doi:10.1152/jn.00125.2003. 12750424

[B36] Grosse P, Cassidy MJ, Brown P EEG-EMG, MEG-EMG and EMG-EMG frequency analysis: physiological principles and clinical applications. Clin Neurophysiol 113: 1523–1531, 2002. doi:10.1016/S1388-2457(02)00223-7. 12350427

[B37] Hale R, Hausselle JG, Gonzalez RV A preliminary study on the differences in male and female muscle force distribution patterns during squatting and lunging maneuvers. Comput Biol Med 52: 57–65, 2014. doi:10.1016/j.compbiomed.2014.06.010. 25016289 PMC4156018

[B38] Hart CB, Giszter SF A neural basis for motor primitives in the spinal cord. J Neurosci 30: 1322–1336, 2010. doi:10.1523/JNEUROSCI.5894-08.2010. 20107059 PMC6633785

[B39] Hermens HJ, Freriks B, Disselhorst-Klug C, Rau G Development of recommendations for SEMG sensors and sensor placement procedures. J Electromyogr Kinesiol 10: 361–374, 2000. doi:10.1016/S1050-6411(00)00027-4. 11018445

[B40] Jackson A, Spinks RL, Freeman TC, Wolpert DM, Lemon RN Rhythm generation in monkey motor cortex explored using pyramidal tract stimulation. J Physiol 541: 685–699, 2002. doi:10.1113/jphysiol.2001.015099. 12068033 PMC2290363

[B41] Jaiser SR, Baker MR, Baker SN Intermuscular coherence in normal adults: variability and changes with age. PLoS One 11: e0149029, 2016. doi:10.1371/journal.pone.0149029. 26901129 PMC4763454

[B42] Jakobi JM, Chilibeck PD Bilateral and unilateral contractions: possible differences in maximal voluntary force. Can J Appl Physiol 26: 12–33, 2001. doi:10.1139/h01-002. 11173667

[B43] Janzen CL, Chilibeck PD, Davison KS The effect of unilateral and bilateral strength training on the bilateral deficit and lean tissue mass in post-menopausal women. Eur J Appl Physiol 97: 253–260, 2006. doi:10.1007/s00421-006-0165-1. 16568338

[B44] Kang N, Roberts LM, Aziz C, Cauraugh JH Age-related deficits in bilateral motor synergies and force coordination. BMC Geriatr 19: 287, 2019. doi:10.1186/s12877-019-1285-x. 31651243 PMC6814115

[B45] Kapreli E, Athanasopoulos S, Papathanasiou M, Van Hecke P, Strimpakos N, Gouliamos A, Peeters R, Sunaert S Lateralization of brain activity during lower limb joints movement. An fMRI study. Neuroimage 32: 1709–1721, 2006. doi:10.1016/j.neuroimage.2006.05.043. 16859927

[B46] Kargo WJ, Giszter SF Rapid correction of aimed movements by summation of force-field primitives. J Neurosci 20: 409–426, 2000. doi:10.1523/JNEUROSCI.20-01-00409.2000. 10627617 PMC6774133

[B47] Keenan KG, Massey WV, Walters TJ, Collins JD Sensitivity of EMG-EMG coherence to detect the common oscillatory drive to hand muscles in young and older adults. J Neurophysiol 107: 2866–2875, 2012. doi:10.1152/jn.01011.2011. 22378168

[B48] Kenville R, Maudrich T, Vidaurre C, Maudrich D, Villringer A, Nikulin VV, Ragert P Corticomuscular interactions during different movement periods in a multi-joint compound movement. Sci Rep 10: 5021, 2020. doi:10.1038/s41598-020-61909-z. 32193492 PMC7081206

[B49] Kilner JM, Baker SN, Salenius S, Jousmäki V, Hari R, Lemon RN Task-dependent modulation of 15-30 Hz coherence between rectified EMGs from human hand and forearm muscles. J Physiol 516: 559–570, 1999. doi:10.1111/j.1469-7793.1999.0559v.x. 10087353 PMC2269269

[B50] Kisiel-Sajewicz K, Fang Y, Hrovat K, Yue GH, Siemionow V, Sun CK, Jaskólska A, Jaskólski A, Sahgal V, Daly JJ Weakening of synergist muscle coupling during reaching movement in stroke patients. Neurorehabil Neural Repair 25: 359–368, 2011. doi:10.1177/1545968310388665. 21343527

[B51] Koh TJ, Grabiner MD, Clough CA Bilateral deficit is larger for step than for ramp isometric contractions. J Appl Physiol (1985) 74: 1200–1205, 1993. doi:10.1152/jappl.1993.74.3.1200. 8482658

[B52] Kouzaki M, Masani K Postural sway during quiet standing is related to physiological tremor and muscle volume in young and elderly adults. Gait Posture 35: 11–17, 2012. doi:10.1016/j.gaitpost.2011.03.028. 21855345

[B53] Laine CM, Valero-Cuevas FJ Intermuscular coherence reflects functional coordination. J Neurophysiol 118: 1775–1783, 2017. doi:10.1152/jn.00204.2017. 28659460 PMC5596118

[B54] Lee TS, Song MY, Kwon YJ Activation of back and lower limb muscles during squat exercises with different trunk flexion. J Phys Ther Sci 28: 3407–3410, 2016. doi:10.1589/jpts.28.3407. 28174462 PMC5276771

[B55] Levine AJ, Hinckley CA, Hilde KL, Driscoll SP, Poon TH, Montgomery JM, Pfaff SL Identification of a cellular node for motor control pathways. Nat Neurosci 17: 586–593, 2014. doi:10.1038/nn.3675. 24609464 PMC4569558

[B56] Magnus CR, Farthing JP Greater bilateral deficit in leg press than in handgrip exercise might be linked to differences in postural stability requirements. Appl Physiol Nutr Metab 33: 1132–1139, 2008. doi:10.1139/H08-101. 19088771

[B57] McAuley JH, Rothwell JC, Marsden CD Frequency peaks of tremor, muscle vibration and electromyographic activity at 10 Hz, 20 Hz and 40 Hz during human finger muscle contraction may reflect rhythmicities of central neural firing. Exp Brain Res 114: 525–541, 1997. doi:10.1007/PL00005662. 9187289

[B58] McManus L, Hu X, Rymer WZ, Suresh NL, Lowery MM Muscle fatigue increases beta-band coherence between the firing times of simultaneously active motor units in the first dorsal interosseous muscle. J Neurophysiol 115: 2830–2839, 2016. doi:10.1152/jn.00097.2016. 26984420 PMC4922605

[B59] Mehls K, Grubbs B, Jin Y, Coons J Electromyography comparison of sex differences during the back squat. J Strength Cond Res. In press. doi:10.1519/JSC.0000000000003469. 32032232

[B60] Merletti R, Di Torino P Standards for reporting EMG data. J Electromyogr Kinesiol 9: 3–4, 1999.

[B61] Mima T, Hallett M Corticomuscular coherence: a review. J Clin Neurophysiol 16: 501–511, 1999. doi:10.1097/00004691-199911000-00002. 10600018

[B62] Mohr M, Nann M, von Tscharner V, Eskofier B, Nigg BM Task-dependent intermuscular motor unit synchronization between medial and lateral vastii muscles during dynamic and isometric squats. PLoS One 10: e0142048, 2015. doi:10.1371/journal.pone.0142048. 26529604 PMC4631473

[B63] Mohr M, Schön T, von Tscharner V, Nigg BM Intermuscular coherence between surface emg signals is higher for monopolar compared to bipolar electrode configurations. Front Physiol 9: 566, 2018. doi:10.3389/fphys.2018.00566. 29867587 PMC5966566

[B64] Mozaffarian D, Benjamin EJ, Go AS, Arnett DK, Blaha MJ, Cushman M, Das SR, de Ferranti S, Després JP, Fullerton HJ, Howard VJ, Huffman MD, Isasi CR, Jiménez MC, Judd SE, Kissela BM, Lichtman JH, Lisabeth LD, Liu S, Mackey RH, Magid DJ, McGuire DK, Mohler ER III, Moy CS, Muntner P, Mussolino ME, Nasir K, Neumar RW, Nichol G, Palaniappan L, Pandey DK, Reeves MJ, Rodriguez CJ, Rosamond W, Sorlie PD, Stein J, Towfighi A, Turan TN, Virani SS, Woo D, Yeh RW, Turner MB; Writing Group Members; American Heart Association Statistics Committee; Stroke Statistics Subcommittee Heart disease and stroke statistics-2016 update: a report from the American Heart Association. Circulation 133: e38–e360, 2016. doi:10.1161/CIR.0000000000000350. 26673558

[B65] Myer GD, Kushner AM, Brent JL, Schoenfeld BJ, Hugentobler J, Lloyd RS, Vermeil A, Chu DA, Harbin J, McGill SM The back squat: a proposed assessment of functional deficits and technical factors that limit performance. Strength Condit J 36: 4–27, 2014. doi:10.1519/SSC.0000000000000103. 25506270 PMC4262933

[B66] Myers LJ, Lowery M, O’Malley M, Vaughan CL, Heneghan C, St Clair Gibson A, Harley YX, Sreenivasan R Rectification and non-linear pre-processing of EMG signals for cortico-muscular analysis. J Neurosci Methods 124: 157–165, 2003. doi:10.1016/S0165-0270(03)00004-9. 12706845

[B67] Nelson DL, Cipriani DJ, Thomas JJ Physical therapy and occupational therapy: partners in rehabilitation for persons with movement impairments. Occup Ther Health Care 15: 35–57, 2002. doi:10.1080/J003v15n03_03. 23952022

[B68] Neto OP, Christou EA Rectification of the EMG signal impairs the identification of oscillatory input to the muscle. J Neurophysiol 103: 1093–1103, 2010. doi:10.1152/jn.00792.2009. 20032241 PMC2822682

[B69] Nguyen HB, Lee SW, Harris-Love ML, Lum PS Neural coupling between homologous muscles during bimanual tasks: effects of visual and somatosensory feedback. J Neurophysiol 117: 655–664, 2017. doi:10.1152/jn.00269.2016. 27852730 PMC5288480

[B70] Noble JW, Eng JJ, Boyd LA Bilateral motor tasks involve more brain regions and higher neural activation than unilateral tasks: an fMRI study. Exp Brain Res 232: 2785–2795, 2014. doi:10.1007/s00221-014-3963-4. 24770862 PMC4486387

[B71] Omlor W, Patino L, Hepp-Reymond M-C, Kristeva R Gamma-range corticomuscular coherence during dynamic force output. Neuroimage 34: 1191–1198, 2007. doi:10.1016/j.neuroimage.2006.10.018. 17182258

[B72] Pizzamiglio S, De Lillo M, Naeem U, Abdalla H, Turner DL High-frequency intermuscular coherence between arm muscles during robot-mediated motor adaptation. Front Physiol 7: 668, 2017. doi:10.3389/fphys.2016.00668. 28119620 PMC5220015

[B73] Poston B, Danna-Dos Santos A, Jesunathadas M, Hamm TM, Santello M Force-independent distribution of correlated neural inputs to hand muscles during three-digit grasping. J Neurophysiol 104: 1141–1154, 2010. doi:10.1152/jn.00185.2010. 20505123 PMC2934922

[B74] Reyes A, Laine CM, Kutch JJ, Valero-Cuevas FJ Beta band corticomuscular drive reflects muscle coordination strategies. Front Comput Neurosci 11: 17, 2017. doi:10.3389/fncom.2017.00017. 28420975 PMC5378725

[B75] Roopun AK, Middleton SJ, Cunningham MO, LeBeau FE, Bibbig A, Whittington MA, Traub RD A beta2-frequency (20–30 Hz) oscillation in nonsynaptic networks of somatosensory cortex. Proc Natl Acad Sci USA 103: 15646–15650, 2006. doi:10.1073/pnas.0607443103. 17030821 PMC1592532

[B76] Rosenberg JR, Amjad AM, Breeze P, Brillinger DR, Halliday DM The Fourier approach to the identification of functional coupling between neuronal spike trains. Prog Biophys Mol Biol 53: 1–31, 1989. doi:10.1016/0079-6107(89)90004-7. 2682781

[B77] Seidler RD, Bernard JA, Burutolu TB, Fling BW, Gordon MT, Gwin JT, Kwak Y, Lipps DB Motor control and aging: links to age-related brain structural, functional, and biochemical effects. Neurosci Biobehav Rev 34: 721–733, 2010. doi:10.1016/j.neubiorev.2009.10.005. 19850077 PMC2838968

[B78] Semmler JG, Ebert SA, Amarasena J Eccentric muscle damage increases intermuscular coherence during a fatiguing isometric contraction. Acta Physiol (Oxf) 208: 362–375, 2013. doi:10.1111/apha.12111. 23621345

[B79] Semmler JG, Kornatz KW, Dinenno DV, Zhou S, Enoka RM Motor unit synchronisation is enhanced during slow lengthening contractions of a hand muscle. J Physiol 545: 681–695, 2002. doi:10.1113/jphysiol.2002.026948. 12456843 PMC2290686

[B80] Semmler JG, Kornatz KW, Meyer FG, Enoka RM Diminished task-related adjustments of common inputs to hand muscle motor neurons in older adults. Exp Brain Res 172: 507–518, 2006. doi:10.1007/s00221-006-0367-0. 16489433

[B81] Singh A, Tetreault L, Kalsi-Ryan S, Nouri A, Fehlings MG Global prevalence and incidence of traumatic spinal cord injury. Clin Epidemiol 6: 309–331, 2014. doi:10.2147/CLEP.S68889. 25278785 PMC4179833

[B82] Singh RS, Prakash H Phase-coherence and amplitude-coherence. Acta Phys Pol B 31: 2075–2084, 2000.

[B83] Spedden ME, Jensen P, Terkildsen CU, Jensen NJ, Halliday DM, Lundbye-Jensen J, Nielsen JB, Geertsen SS The development of functional and directed corticomuscular connectivity during tonic ankle muscle contraction across childhood and adolescence. Neuroimage 191: 350–360, 2019. doi:10.1016/j.neuroimage.2019.02.054. 30818025

[B84] Stegeman DF, van de Ven WJ, van Elswijk GA, Oostenveld R, Kleine BU The alpha-motoneuron pool as transmitter of rhythmicities in cortical motor drive. Clin Neurophysiol 121: 1633–1642, 2010. doi:10.1016/j.clinph.2010.03.052. 20434397

[B85] Terry K, Griffin L How computational technique and spike train properties affect coherence detection. J Neurosci Methods 168: 212–223, 2008. doi:10.1016/j.jneumeth.2007.09.014. 17976736 PMC2268650

[B86] Thiele RM, Conchola EC, Palmer TB, DeFreitas JM, Thompson BJ The effects of a high-intensity free-weight back-squat exercise protocol on postural stability in resistance-trained males. J Sports Sci 33: 211–218, 2015. doi:10.1080/02640414.2014.934709. 24998744

[B87] Ushiyama J, Takahashi Y, Ushiba J Muscle dependency of corticomuscular coherence in upper and lower limb muscles and training-related alterations in ballet dancers and weightlifters. J Appl Physiol (1985) 109: 1086–1095, 2010. doi:10.1152/japplphysiol.00869.2009. 20689093

[B88] van Asseldonk EH, Campfens SF, Verwer SJ, van Putten MJ, Stegeman DF Reliability and agreement of intramuscular coherence in tibialis anterior muscle. PLoS One 9: e88428, 2014. doi:10.1371/journal.pone.0088428. 24520387 PMC3919778

[B89] von Tscharner V Task dependent synchronization of motor units of the medial gastrocnemius muscle revealed in EMG-currents. J Exerc Sports Orthop 1: 1–7, 2014. doi:10.15226/2374-6904/1/1/00105.

[B90] von Tscharner V, Ullrich M, Mohr M, Comaduran Marquez D, Nigg BM Beta, gamma band, and high-frequency coherence of EMGs of vasti muscles caused by clustering of motor units. Exp Brain Res 236: 3065–3075, 2018. doi:10.1007/s00221-018-5356-6. 30128624

[B91] Walker S, Avela J, Wikgren J, Meeusen R, Piitulainen H, Baker SN, Parviainen TM Aging and strength training influence knee extensor intermuscular coherence during low- and high-force isometric contractions. Front Physiol 9: 1933, 2019. doi:10.3389/fphys.2018.01933. 30728782 PMC6351450

[B92] Welch P The use of fast Fourier transform for the estimation of power spectra: a method based on time averaging over short, modified periodograms. IEEE Trans Audio Electroacoust 15: 70–73, 1967. doi:10.1109/TAU.1967.1161901.

[B93] Whitall J, Waller SM, Sorkin JD, Forrester LW, Macko RF, Hanley DF, Goldberg AP, Luft A Bilateral and unilateral arm training improve motor function through differing neuroplastic mechanisms: a single-blinded randomized controlled trial. Neurorehabil Neural Repair 25: 118–129, 2011. doi:10.1177/1545968310380685. 20930212 PMC3548606

[B94] Witham CL, Riddle CN, Baker MR, Baker SN Contributions of descending and ascending pathways to corticomuscular coherence in humans. J Physiol 589: 3789–3800, 2011. doi:10.1113/jphysiol.2011.211045. 21624970 PMC3171886

[B95] Witham CL, Wang M, Baker SN Corticomuscular coherence between motor cortex, somatosensory areas and forearm muscles in the monkey. Front Syst Neurosci 4: 38, 2010. doi:10.3389/fnsys.2010.00038. 20740079 PMC2927302

[B96] Yao B, Salenius S, Yue GH, Brown RW, Liu JZ Effects of surface EMG rectification on power and coherence analyses: an EEG and MEG study. J Neurosci Methods 159: 215–223, 2007. doi:10.1016/j.jneumeth.2006.07.008. 16949676

[B97] Yavuz HU, Erdag D Kinematic and electromyographic activity changes during back squat with submaximal and maximal loading. Appl Bionics Biomech 2017: 9084725, 2017. doi:10.1155/2017/9084725. 28546738 PMC5435978

